# Markers of Inflammation Associated with Plaque Progression and Instability in Patients with Carotid Atherosclerosis

**DOI:** 10.1155/2015/718329

**Published:** 2015-04-16

**Authors:** Enrico Ammirati, Francesco Moroni, Giuseppe Danilo Norata, Marco Magnoni, Paolo G. Camici

**Affiliations:** ^1^Cardiothoracic Department, San Raffaele Scientific Institute and Vita-Salute San Raffaele University, Via Olgettina 58-60, 20132 Milan, Italy; ^2^Cardiovascular and Thoracic Department, Niguarda Ca' Granda Hospital, Milan, Italy; ^3^Departement of Pharmacological and Biomolecular Sciences, Università degli Studi di Milano, Milan, Italy; ^4^Center for the Study of Atherosclerosis, Bassini Hospital, Cinisello Balsamo, Italy; ^5^Blizard Institute, Queen Mary University, London, UK

## Abstract

Atherosclerosis is the focal expression of a systemic disease affecting medium- and large-sized arteries, in which traditional cardiovascular risk factor and immune factors play a key role. It is well accepted that circulating biomarkers, including C-reactive protein and interleukin-6, reliably predict major cardiovascular events, including myocardial infarction or death. However, the relevance of biomarkers of systemic inflammation to atherosclerosis progression in the carotid artery is less established. The large majority of clinical studies focused on the association between biomarkers and subclinical atherosclerosis, that is, carotid intima-media thickening (cIMT), which represents an earlier stage of the disease. The aim of this work is to review inflammatory biomarkers that were associated with a higher atherosclerotic burden, a faster disease progression, and features of plaque instability, such as inflammation or neovascularization, in patients with carotid atherosclerotic plaque, which represents an advanced stage of disease compared with cIMT. The association of biomarkers with the occurrence of cerebrovascular events, secondary to carotid plaque rupture, will also be presented. Currently, the degree of carotid artery stenosis is used to predict the risk of future cerebrovascular events in patients affected by carotid atherosclerosis. However, this strategy appears suboptimal. The identification of suitable biomarkers could provide a useful adjunctive criterion to ensure better risk stratification and optimize management.

## 1. Introduction

Atherosclerotic disease of the carotid arteries appears to be highly prevalent in the ageing population. According to recent epidemiological surveys, up to 5% of women and 12% of men over the age of 80 suffer from asymptomatic moderate (i.e., reduction in diameter between 50% and 70%) atherosclerotic carotid artery stenosis, while 1% and 3%, respectively, suffer from severe carotid stenosis, defined as a reduction in diameter of ≥70% [1]. It is well recognized that the presence of atherosclerotic disease in the carotid arteries poses a substantial risk of ipsilateral cerebrovascular events, with reported annual ischemic stroke rates ranging from 0.35% to 1.3% in asymptomatic patients with moderate stenosis [2, 3] and from 0.5% to approximately 5% for severe asymptomatic carotid artery stenosis [2, 4]. Around 20% of ischemic strokes appear to originate from carotid plaques [5], mainly due to an arterioarterial embolization [6], calling for the institution of appropriate management strategies aimed at effectively minimizing risk. Current guidelines recommend surgical or endovascular revascularization in patients bearing an asymptomatic carotid artery stenosis of ≥60% and with a life expectancy of more than 5 years [7]. The appropriateness of such indication is, however, challenged by the evidence provided by randomized controlled trials and meta-analyses that have shown no benefit of revascularization over optimal medical therapy in the management of asymptomatic carotid stenosis [8, 9]. Current risk stratification strategies appear to be inadequate in reliably identifying individuals who may benefit from aggressive treatment [10, 11].

Apart from the risk of stroke, the presence of severe asymptomatic carotid artery stenosis was reproducibly shown to be associated with cognitive decline [12, 13]. The pathogenic mechanism appears to involve both chronic hypoperfusion [14] and microembolic events [15]. The possibility of developing mild cognitive impairment may thus be taken into account when choosing the appropriate management strategy for asymptomatic carotid artery stenosis.

Several serum inflammatory markers have been proposed as tools for risk assessment in patients bearing atherosclerotic lesions of the carotid artery. Among them, notable examples include fibrinogen, serum amyloid A (SAA), interleukin-6 (IL-6), and lipoprotein-associated phospholipase A2 (Lp-PLA2), which was recently approved by United States Food and Drugs Administration as a predictor of ischemic stroke [16, 17]. The most widely used in current clinical practice however remains high sensitivity C-reactive protein (hs-CRP), which was shown to reproducibly predict the risk of stroke in several large epidemiological studies, including the Physicians' Health Study [18], Women's Health Study [19], and the Framingham Heart Study [20]. In addition, the* in vivo* identification of plaque features predictive of progression or instability would be advantageous in establishing the appropriate clinical management, including imaging follow-up and therapeutic interventions. Serum biomarkers reflecting the activity of biological processes involved in plaque growth or destabilization may provide great help for this purpose.

Of note, early atherosclerosis, measured as carotid intima-media thickness (cIMT), correlates with the risk of cardiovascular events in the general population. However, an association between changes in cIMT and the risk of cardiovascular events (including myocardial infarction, stroke, vascular death, or a combination of these) remained unproven in a large survey of more than 36000 subjects from the general population [21]. Despite this, patients with the highest “inflammatory load” had the greater cIMT progression [22], further underlining the relevance of serum inflammatory biomarkers as surrogates that could reflect processes associated with atherosclerotic disease progression. In the present work we critically review the major studies identifying inflammatory biomarkers associated with atherosclerotic disease progression, features of plaque vulnerability (including inflammation and neovascularization), or cerebrovascular symptoms, expanding and updating the concepts expressed by Hermus et al. in a previous article [23].  Table 1 summarizes the current available evidence on the association between carotid atherosclerosis and biomarkers.

*Search Strategy*. We searched PubMed electronic database, limiting the search to original research published between 1990 and December 2014. The key words used for the search were* carotid stenosis and markers of inflammation*,* carotid stenosis and T cells*,* carotid stenosis and monocytes*,* carotid plaque neovascularization and markers of inflammation*,* carotid plaque vulnerability and markers of inflammation.*

## 2. Inflammatory Biomarkers and Carotid Artery Plaque

### 2.1. Markers of Inflammation and Degree of Stenosis

Identifying patients at risk of bearing an asymptomatic carotid artery stenosis appears of great importance for the implementation of reliable yet cost-effective ultrasonographic screening programs. Serum markers of inflammation may appropriately serve this purpose. As mentioned earlier, CRP is commonly used for cardiovascular risk evaluation. Recent reports have shown that higher serum hs-CRP levels are in fact able to predict the presence of carotid artery plaque [24, 25] but do not associate with the degree of stenosis the lesions determine [24]. These data contrast with previous reports on larger populations, which failed to demonstrate a similar association. In particular, Halvorsen and colleagues could not establish an association between CRP and presence of carotid plaque in a cross-sectional study involving 5341 individuals, both males and females [26], and similarly Chapman et al. obtained a similar result in a cohort of 1111 subjects [27].

The association between the presence and severity of carotid stenosis and other acute phase proteins of the pentraxin superfamily, which includes CRP, such as the long pentraxin-3 (PTX3), is not clear [28, 29]. While PTX3 was shown to be atheroprotective in experimental atherosclerosis [30, 31], preliminary data in humans suggest that plasma levels of PTX3 are associated with the presence of atherosclerotic plaques and prevalent vascular disease [32]. A subsequent population-based study involving more than 2400 subjects, however, showed that PTX3 is not a predictor of incident cardiovascular events [33].

Other markers have been explored, including fibrinogen, which was more reliably associated with the presence of plaque than CRP [24, 26, 27], sVCAM [25], and erythrocyte sedimentation rate (ESR) [24], all of which showed some association with carotid atherosclerosis. Of great interest was the fact that IL-6, a master proinflammatory cytokine, was reproducibly shown to be higher in the serum of subjects bearing an asymptomatic carotid stenosis with respect to controls [24, 25, 27]. S100A12, a calcium binding protein involved in inflammatory signaling, was also shown to be elevated in patients bearing carotid atherosclerotic lesions [34]. Notably, none of these markers was shown to correlate with the degree of stenosis. On the other hand, serum levels of TNF*α* and L-selectin, a lectin type adhesion molecule expressed on leukocytes surface, were shown to associate with larger plaque size estimated by ultrasound imaging in a cohort of 1016 subjects [35].

### 2.2. Markers of Inflammation and Plaque Instability

Plaques at higher risk of causing an acute atherothrombotic or atheroembolic event, including stroke and acute myocardial infarction, that is, unstable plaques, share some distinctive features, such as a thinner fibrous cap overlying a large necrotic core or a strong intraplaque inflammatory reaction [36, 37]. The detection of potentially unstable atherosclerotic lesions using biomarkers would bring obvious advantages in establishing an appropriate treatment plan in patients with an asymptomatic carotid stenosis. Features of plaque instability as evaluated by magnetic resonance imaging (MRI), that is, signal hypointensity in T1 weighted images, were shown to correlate with the upregulation of several proinflammatory molecules, such as the cytokines IL-6 and TNF*α*, the endothelial activation markers E-cadherin and VCAM-1, and the inflammatory markers hs-CRP and, most notably, PTX3 [38]. A recent report by Willems et al. failed to show an association between serum concentration of ST2, a member of IL1 receptor family, and a vulnerable plaque phenotype, as determined histologically [39]. Sugioka and colleagues have recently shown that serum levels of neopterin, a product of the catabolism of guanosine triphosphate secreted by macrophages upon activation, correlated with the presence of highly complex carotid lesions, suggestive of plaque vulnerability, in patients affected by stable coronary artery disease [40]. In a cohort of 101 patients, Pelisek and colleagues were able to establish an association between histological features of carotid plaque instability and serum levels of circulating matrix metalloproteinase (MMP)-1, MMP-7, tissue inhibitor of matrix protease (TIMP)-1, and IL-8 [41]. MMPs are a class of proteases involved in extracellular matrix degradation, which appear to play a key role in the process of vascular remodeling during the course of vascular disease [42]. The fact that numerous experimental studies suggest that MMPs may be involved in the process of plaque destabilization [43, 44] makes this finding particularly intriguing.

### 2.3. Markers of Inflammation and Neurological Symptoms

The ultimate goal of managing a patient suffering from asymptomatic carotid artery stenosis is, as reiterated above, preventing the occurrence of atheroembolic events leading to ischemic stroke. Studying patients soon after a cerebrovascular event of carotid origin might lead to the identification of serum markers potentially useful in identifying patients at higher risk for recurrence. Clearly, results obtained in the acute phase of disease should, however, be considered critically, since the acute process within the central nervous system may directly influence the concentration of circulating biomarkers, increasing the risk of spurious associations. Again, the analysis of hs-CRP has led to contradictory results, making it rather unreliable for the identification of patients deserving an aggressive management [24, 25, 45, 46]. IL-6, on the other hand, was shown to be more reproducibly associated with the presence of a symptomatic carotid stenosis, but the results are far from being conclusive [24, 25, 45]. Among other inflammatory markers explored, the soluble form of CD36, a macrophage scavenger receptor involved in LDL uptake, was shown to correlate with the presence of symptoms and ultrasound features of plaque vulnerability in a cohort of 62 patients, 31 of which had suffered from stroke in the previous 6 months [47]. Interestingly, asymptomatic and symptomatic patients did not differ in terms of CRP, glycosylated hemoglobin, or lipid profile [47]. Plasma soluble urokinase plasminogen activator receptor (suPAR) is the soluble form of the cell-surface urokinase plasminogen activator receptor, released by endothelial and immune cells by proteolytic cleavage in an inflammatory environment [48]. Plasma levels of suPAR were higher in symptomatic patients than in patients bearing an asymptomatic carotid artery stenosis in a recent report on 162 patients [49]. In addition, suPAR was higher in patients suffering from stroke or TIA than symptomatic patients in which carotid atherosclerosis had manifested as amaurosis fugax [50]. Another soluble form of a receptor, the receptor for advanced glycosylation end products (RAGE), which is involved in pattern recognition, was shown to associate with the presence of symptoms in patients affected by carotid artery atherosclerosis, albeit in a small study comprising only 29 patients [51]. Consistent with its association with plaque features of instability, MMP-7 was shown to be elevated in the sera of patients who had a stroke from 2 to 6 months prior to the analysis [52].

## 3. Systemic versus Local Inflammation

Inflammation of blood vessel wall is a critical component of atherosclerosis and brings about several pathological changes such as edema, vasa vasorum dilation and proliferation, and immune cells infiltration [53]. The knowledge of the biological basis of these processes has led to the development of novel noninvasive imaging technique able to disclose them* in vivo* through the use of appropriately designed molecular probes [36, 54]. In this respect, positron emission tomography (PET) using the radiolabelled glucose analogue 18F-fluorodeoxyglucose (FDG) is the most widely used imaging tool, allowing the detection of high metabolic activity in inflamed plaques [54]. Most interestingly, plaque inflammation is associated with atherothrombotic manifestation, but its relation to serum biomarkers is unclear. In a recently reported post hoc analysis of 130 patients participating in the dal-PLAQUE study, baseline FDG uptake in the most diseased carotid segments positively correlated with blood myeloperoxidase, an effector molecule secreted by granulocytes upon stimulation, in patients suffering from coronary artery disease on statin therapy [55]. In the same cohort, blood levels of IL-6 positively correlated with FDG target-to-background ratio (TBR) in the most diseased carotid segments [56]. Interestingly, hs-CRP, soluble P-selectin and E-selectin, sVICAM1, and MMP-3 and MMP-9 did not correlate with FDG uptake in the carotid artery [55]. Interestingly however in an earlier report by Rudd on 40 patients and colleagues FDG uptake in the carotid artery significantly correlated with serum MMP-9 levels, while a negative correlation was established between FDG TBR in the carotid artery and serum plasminogen activator inhibitor 1 (PAI1) [57]. Again, hs-CRP did not correlate with plaque metabolic activity [57]. The lack of correlation between hs-CRP and plaque inflammation was recently confirmed by immune-pathological analysis, that is, the current gold standard, of 160 carotid endarterectomy specimens [58].

Although not strictly considered inflammatory markers due to their principal function as mediators of extracellular matrix remodelling [59], MMPs are gaining a growing interest in the field of atherosclerosis. A recent report suggested that they may become themselves the target of radiolabelled molecular probes for the detection of vulnerable plaques [60].

## 4. Circulating Lipids, Inflammation, and Plaque Progression

Plasma low-density lipoprotein cholesterol (LDL-C) level is considered one of the most important risk factors for atherosclerotic plaque instability and several emerging pharmacological approaches are aimed at decreasing vascular inflammation and increasing plaque stability by reducing LDL-C levels [61]. In spite of this, many individuals with evidence of atherosclerotic disease have LDL-C within the normal range, although they might have an altered pattern of lipoproteins subfractions. The concentration of small dense low-density lipoprotein (sdLDL) has been associated with increased cardiovascular risk and with the progression of coronary and carotid atherosclerosis in case-control and prospective studies [62–64]. The lipoprotein cholesterol profile and the LDL floatation rate (LDL-RF) were directly and significantly correlated with weight, body mass index, waist, hip, waist/hip ratio, triglycerides, fasting glycaemia, and cIMT and inversely related to high-density lipoprotein cholesterol (HDL-C) [65]. Among the lipoprotein subclasses, triglyceride-rich lipoproteins (TGRL) and sdLDL were shown to independently predict the entity of cIMT and were associated with a proinflammatory activation of peripheral mononuclear cells and endothelial cells [65]. These findings suggest that cholesterol or triglycerides content in lipoproteins could mark specific lipoprotein subclasses with specific atherogenic and proinflammatory properties [66, 67]. The presence of such specific lipoprotein subsets could be also the result of the activation of lipoprotein remodelling enzymes, which occurs during inflammation. For example, the activation of innate immune response results in the reduction of plasma HDL-C levels but also in the remodelling of high-density lipoprotein (HDL) which becomes enriched in proinflammatory mediators and becomes dysfunctional [68–70].

Impaired HDL functionality was also observed in immune-inflammatory and autoimmune diseases [69, 71] and was associated with altered lymphocyte subsets distribution and increased cIMT and atherosclerotic plaques [72]. Another key above-mentioned molecule generated during inflammatory processes is the Lp-PLA2, which is associated with increased risk of cardiovascular events [17]. Lp-PLA2 mediates the formation of bioactive mediators (lysophosphatidyl choline and oxidized nonesterified fatty acids) known to elicit several deleterious inflammatory responses involved in the pathobiology of atherosclerosis [73]. Lysophosphatidyl, for example, choline, serves as a potent chemoattractant for monocytes, resulting in foam cell accumulation within the arterial wall [73]. Despite these observations the role of Lp-PLA2, as well as that of the secretory PLA2 (sPLA2), remains enigmatic. In elderly subjects sPLA2 levels, but not Lp-PLA2 levels, were independently associated with atherosclerotic plaques and outcome [74]. Data from the above-mentioned dal-PLAQUE study demonstrated that baseline Lp-PLA2 mass correlated with TBR of FDG uptake in the aorta but not in the carotid artery [55].

A new intriguing molecule related to inflammation and lipid metabolism, the fatty acid binding protein 4 (FABP4 or aP2 in mice), has been identified as a key regulator of core aspects of cardiometabolic disorders, including lipotoxic endoplasmic reticulum stress in macrophages, and macrophage cholesterol trafficking and associated inflammation [75]. In a large study the effects of low-expression variant of FABP4 were examined at population level (*n* = 7491) and in group of patients with advanced carotid atherosclerosis (*n* = 92) and myocardial infarction (*n* = 3432) [76]. The authors found that the low-expression variant was associated with decreased total cholesterol levels. Furthermore, the carriers of the allelic variant associated with obesity also showed reduced cIMT thickness and lower prevalence of carotid plaques [76]. In another study, plasma levels of FABP4 were also evaluated in relation to symptoms in 59 patients with carotid plaques and 202 having suffered from stroke of another origin [77]. FABP4 levels were higher in patients with carotid atherosclerosis, both systemically and within the atherosclerotic lesion, than in patients having suffered from a stroke from another origin, with particularly high mRNA levels in carotid plaques from patients with the most recent symptoms [77]. Furthermore, FABP4 correlated with the cell-surface markers of monocyte/macrophage lineage CD36, CD68, and CD163 as well as with the presence of CD4-positive T cells in the plaque [78].

Finally, alterations in plasma lipid profile and inflammation are often presenting in deep relation to metabolic status, which could mark the presence of insulin resistance and/or diabetes. While the discussion of the latter is beyond the scope of the present review, it is important to mention that metabolic inflammation could be associated with changes in the circulating profile of key cytokines such as adipokines. To this extent, resistin, leptin, and adiponectin could mark the altered metabolic status which is associated with dyslipidaemia, systemic inflammation, and vascular disease [79–83].

## 5. Circulating Leukocytes and Plaque Progression

White blood cells constitute the effector arm of the immune system, attending to both immune surveillance and prompt response to tissue damage. Several cell types are found among white blood cells, each with a different function and differentially activated by specific stimuli. In the last decades, forefront technologies such as polychromatic flow cytometry have allowed the identification of thousands of leukocytes subsets [84], giving the chance to identify alterations in the face of normal white blood cells counts. A careful analysis of circulating leukocytes may thus provide a valuable tool to evaluate the inflammatory and immune status of the patient [85]: using this approach circulating white blood cells may well serve as biomarkers. Among the different cells found to be altered in patients with atherosclerosis, mononuclear cells, both lymphocytes [85] and monocytes [86] subpopulations have been most frequently implicated. In addition, gene expression profiling of leukocytes may provide a valuable tool for the identification of specific types of inflammatory response associated with plaque progression or vulnerability. Interestingly, to date only few studies analyzed the association between circulating immune cells and advanced carotid atherosclerotic disease. In fact, most studies concerned with the carotid artery focused on subclinical atherosclerotic disease, such as cIMT. Population-based studies have demonstrated the association between the presence of plaque and total white blood cells [24, 26] and monocytes counts [24, 27]. A recent report on 853 asymptomatic individuals affected by atherosclerosis failed to demonstrate any association between the severity of stenosis or plaque progression at 6 months and white blood cells subtypes [87]. Interestingly, however, neutrophil counts conferred a relative risk of cardiovascular death of 1.9–2.4 in patients bearing a stenosis of >50% [87]. Neutrophil count was also shown to strongly associate with the presence of microembolism detected by transcranial Doppler ultrasound in 60 recently symptomatic patients [88].

Monocytes of recently symptomatic patients were shown to bear signs of activations: in particular, they were found to express high concentrations in the adhesion molecules CD11b and thrombospondin 1 [89]. Interestingly, these same markers were shown to correlate with the presence of platelet-monocyte aggregates [89], which appear to be involved in the pathogenesis of atherosclerosis and atherothrombosis [85, 90]. However, platelet-monocyte aggregates were not shown to associate with the presence of microembolic signals on transcranial Doppler ultrasound in 16 symptomatic and 30 asymptomatic patients affected by carotid atherosclerosis [91]. A subsequent report by Sternberg and colleagues failed to demonstrate an association between higher concentrations of activated monocytes, identified as CD14+CD68+ cells, and the presence of carotid artery atherosclerosis [92]. Notably, patients bearing a carotid plaque had higher activation of T and B lymphocytes, identified as CD3+HLA-DR+ and CD20+CD69+, respectively, and higher levels of MMP-9 expression, assessed through mRNA analysis, in peripheral blood mononuclear cells [92]. Consistently, a study by Martin-Ventura et al. showed an increased expression of CD74 (invariant polypeptide of major histocompatibility complex) in peripheral mononuclear blood of patients with carotid artery stenosis [93]. In a recent report on 700 patients selected from the cardiovascular cohort of the Malmö Diet and Cancer study, the percentage of circulating CD19+CD86+ B cells was shown to correlate with higher degree of carotid artery stenosis [94]. In addition, patients with higher percentages of the same B cells subset were prospectively shown to be at higher risk for stroke [94]. Leukocyte telomere length (LTL) is an important determinant of telomere function and cellular replicative capacity, which were both associated with cardiovascular diseases [95, 96]. An association between telomere shortening (TS) and both the progression of atherosclerosis and the incidence of cardiovascular events (CVEs) was recently shown [97]. After adjusting for classical cardiovascular disease risk factors (age, gender, smoking, physical activity, alcohol consumption, systolic blood pressure, glucose levels, lipid profile, and therapies), TS was associated with increased progression of cIMT [97]. Finally, subjects in whom LTL shortened over time showed an increased risk of incident CVE, compared to those in whom LTL lengthened [97]. These data indicate that leukocyte TS is associated with increased risk of subclinical carotid vascular damage and increased incidence of CVEs beyond classical cardiovascular disease risk factors in the general population, whereas LTL lengthening is protective, further connecting leukocytes counts, function, and senescence with atherosclerotic disorders.

## 6. Circulating Markers of Inflammation and Intima-Media Thickness

As mentioned above, thickening of tunica intima and media of the carotid artery, that is, cIMT, is currently considered an early sign of preclinical atherosclerosis. Accordingly, the presence of increased cIMT was shown to increase the risk of future CVEs [98] and to correlate with the presence of traditional cardiovascular risk factors [99]. However, pathogenesis of cIMT is only incompletely understood [100, 101] and its relationship with carotid artery plaque is not fully defined. For these reasons, a thorough discussion of the association between inflammatory biomarkers and cIMT does not appear completely relevant to the purpose of the present work. Due to the extreme interest in the subject grown in the last few years, however, we will briefly discuss some of the most interesting results in the field. In the aforementioned study by Chapman et al., plasma levels of IL-6, fibrinogen, and monocytes counts were shown to associate with cIMT [27]. However, the significance of association was lost upon correction for conventional risk factors [27]. In a population-based sample of 3092 subjects older than 55 years of age, high levels of hs-CRP were associated with higher cIMT [102]. Interestingly, no significant differences in hs-CRP levels were found between patients with subclinical atherosclerosis and patients bearing carotid artery plaques [102]. A recently published meta-analysis comprising 20 studies and 49097 patients showed a significant association between cIMT and serum levels of hs-CRP, fibrinogen, and leukocytes count [22]. Interestingly, MMPs were once again found to associate with vascular disease in the carotid artery: in a report by Orbe and colleague on 400 otherwise healthy subjects, cIMT was shown to positively correlate with serum levels of MMP-10 [103]. When circulating cells are concerned, a recent report on 912 patients from the Multiethnic Study of Atherosclerosis (MESA) demonstrated a higher number of memory T cells and lower amounts of naïve T cells in subjects with higher cIMT [104]. Accordingly, higher amounts of CD3+CD4+CD45RA−CD45RO+CCR7− effector memory T cells were found to associate with cIMT [105]. In 622 healthy volunteers CD16+ monocytes were shown to associate both with body mass index (BMI) and with cIMT [106]. The relevance of LTL to cIMT was already discussed above [97].

## 7. Markers of Inflammation and Neoangiogenesis

Progression of atherosclerotic disease with plaque enlargement results in intraplaque hypoxia, which contributes to local inflammation and triggers local neovascularization [107]. The presence of neovessels within atherosclerotic lesions was shown not only to promote plaque growth, but also to contribute to its vulnerability [108]. Thus,* in vivo* identification of atherosclerotic lesion neovascularization appears to be a suitable approach for the identification of high-risk plaques. Novel noninvasive imaging strategies, in particular contrast-enhanced ultrasound (CEUS), allow the identification of carotid artery plaque neovessels by dynamically assessing plaque uptake of microsized gas-filled particles known as microbubbles [109, 110]. Currently only few studies assessed the association between the presence of carotid artery plaque neovascularization and circulating biomarkers. A recent report by Qian and colleagues on 40 patients suggests that histologically determined carotid artery vascularization associates with serum levels of CD146 and adhesion molecule of the immunoglobulin superfamily which is implicated in leukocytes extravasation [111]. In another report on 56 patients, the presence of neovessels on carotid plaque surgical specimens positively correlated with serum levels of vascular endothelial growth factor, a master cytokine for angiogenesis and inflammation [112]. Finally, in a recent report Jaipersad and colleagues were able to show a significant correlation between severe plaque neovascularization, detected by CEUS, and circulating levels of classical CD14++CD16−CCR2+ monocytes [113]. In addition, circulating monocytes were shown to express high levels of proangiogenic molecules, such as Tie2 [113].

## 8. Markers of Inflammation and Therapeutic Intervention

To date, several population-based preventive programs aimed at cardiovascular risk reduction were able to substantially abate cardiovascular morbidity and mortality [114, 115]. Most importantly, the introduction of statin therapy was able to reduce cardiovascular mortality by over one-third [116]. Interestingly, aside from statin therapy main effect on cholesterol lowering, statins were shown to reduce the concentration of circulating markers of inflammation [117]. However, a substantial risk needs to be addressed and novel therapeutic targets are needed, alongside novel therapeutic endpoints to assess its efficacy. Assessing intraplaque inflammation and its response to treatment may provide a short term secondary endpoint for clinical studies. In a study by Tang and colleagues, for example, the institution of high dose statin therapy was associated with lowering of carotid artery plaque inflammation assessed through magnetic resonance imaging (MRI) after six and twelve weeks of follow-up [118]. A report by Corti et al. confirms these results and interestingly shows an association between the entity of LDL cholesterol reduction and the disappearance of wall features of inflammation in MRI [119]. In the recently published dal-PLAQUE study, the FDG TBR in the most diseased carotid artery segment was shown to be significantly reduced after 6 months of Dalcetrapib, which modulates cholesteryl ester transfer protein activity to raise high-density lipoprotein cholesterol [120].

## 9. Conclusions

**Figure 1:**
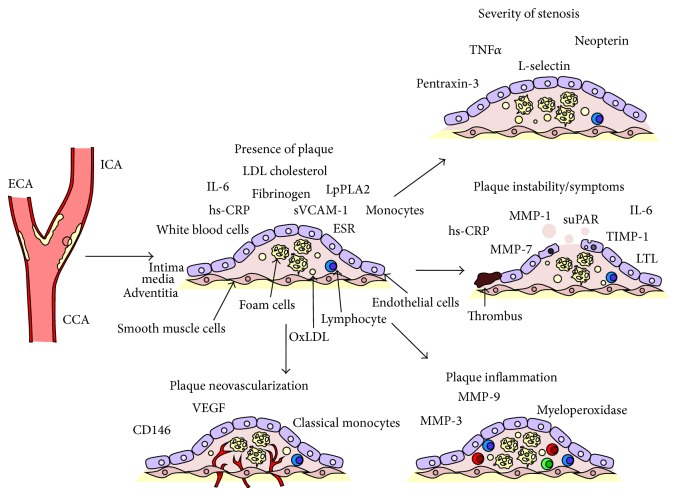
Summary of the main biomarkers associated with different stages and manifestation of carotid atherosclerosis. CCA: common carotid artery; ICA: internal carotid artery; ECA: external carotid artery; IL: interleukin; LDL: low-density lipoprotein; OxLDL: oxidized low-density lipoprotein; ESR: erythrocyte sedimentation rate; hs-CRP: high sensitivity C-reactive protein; sVCAM-1: soluble vascular cell adhesion molecule 1; LpPLA2: lipoprotein-associated phospholipase A2; TNF-*α*: tumour necrosis factor alpha; MMP: matrix metalloproteinase; TIMP-1: tissue inhibitor of matrix proteinases; suPAR: soluble urokinase plasminogen activator receptor; LTL: leukocyte telomere length; VEGF: vascular endothelial growth factor.

Carotid atherosclerosis is highly prevalent in the general population and exerts a high toll in terms of disability and mortality. Currently, the therapeutic management of carotid plaques strongly relies on the severity of stenosis as the primary guide for choosing the appropriate intervention. However, this approach has proved largely unsatisfactory [11]. Inflammation is a basic pathogenic element in the development of atherosclerotic disease and in its manifestation [53]. The detection of the inflammatory and immune profile of the patient may lead to a better stratification of asymptomatic patients, but also of patients at risk for stroke recurrence [121], beyond the simple evaluation of stenosis. In particular, master cytokines such as IL-6 and TNF*α* were shown to reliably predict the presence and the characteristics of the plaque, while novel markers such as the members of the MMP family appear to be implicated in its destabilization. Inflammatory biomarkers may also provide a tool to longitudinally follow patients in order to assess their response to therapy, allowing a better selection of appropriate strategy on the path towards personalized medicine.  Table 2 provides a summary of the studies concerned with the association between inflammatory biomarkers and carotid atherosclerosis while Figure 1 graphically recapitulates the relation between biomarkers and carotid plaque evolution and complications.

## Figures and Tables

**Table 1:** Summary of the main findings on the relation between biomarkers and carotid atherosclerosis.

Marker	IMT	Presence of plaque	Severity of stenosis	Imaging features of vulnerability	Histological features of vulnerability	Symptoms	Imaging features of inflammation	Neovascularization
hs-CRP	++	++− −	−	NA	NA	++− − −	− −	NA
Fibrinogen	+	+++	−	NA	NA	NA	NA	NA
sVCAM-1	NA	+	NA	+	NA	NA	−	NA
ESR	NA	+	−	NA	NA	NA	NA	NA
IL-6	NA	+++	−	+	NA	++−	+	NA
S100A12	NA	+	NA	NA	NA	+	NA	NA
White blood cells	+	++	−	NA	NA	NA	NA	NA
Monocytes	NA	++	−	NA	NA	NA	NA	NA
TNF*α*	NA	NA	+	+	NA	NA	NA	NA
Pentraxin-3	NA	NA	+	NA	NA	NA	NA	NA
L-selectin	NA	NA	+	NA	NA	NA	NA	NA
Neopterin	NA	NA	+	NA	NA	NA	NA	NA
E-cadherin	NA	NA	NA	+	NA	NA	NA	NA
ST2	NA	NA	NA	NA	+	NA	NA	NA
MMP-1	NA	NA	NA	NA	+	NA	NA	NA
MMP-3	NA	NA	NA	NA	NA	NA	+−	NA
MMP-7	NA	NA	NA	NA	+	+	NA	NA
MMP-9	NA	NA	NA	NA	NA	NA	+−	NA
MMP-10	+	NA	NA	NA	NA	NA	NA	NA
TIMP-1	NA	NA	NA	NA	+	NA	NA	NA
IL-8	NA	NA	NA	NA	+	NA	NA	NA
CD36	NA	NA	NA	NA	NA	+	NA	NA
suPAR	NA	NA	NA	NA	NA	++	NA	NA
s-RAGE	NA	NA	NA	NA	NA	+	NA	NA
Myeloperoxidase	NA	NA	NA	NA	NA	NA	+	NA
Neutrophil count	NA	NA	NA	+	NA	NA	NA	NA
CD3+HLA-DR+ T cells	NA	+	NA	NA	NA	NA	NA	NA
CD20+CD69+ B cells	NA	+	NA	NA	NA	NA	NA	NA
CD19+CD86+ B cells	NA	NA	+	NA	NA	+	NA	NA
Leukocyte telomere length	+	NA	NA	NA	NA	+	NA	NA
Memory T cells	+	NA	NA	NA	NA	NA	NA	NA
CD3+CD4+CD45RA−CD45RO+CCR7− T effector memory cells	+	NA	NA	NA	NA	NA	NA	NA
CD16+ monocytes	+	NA	NA	NA	NA	NA	NA	NA
CD146	NA	NA	NA	NA	NA	NA	NA	+
VEGF	NA	NA	NA	NA	NA	NA	NA	+
CD14++CD16−CCR2+ monocytes	NA	NA	NA	NA	NA	NA	NA	+
Lp-PLA2	NA	+	NA	NA	NA	NA	−	NA
FABP4	NA	NA	NA	NA	NA	+	NA	NA

A plus (+) represents a strong clinical evidence in favor of the association, while a minus (−) represents the failure of a well-designed study to establish an association. NA: not available.

**Table 2:** Summary of the main studies concerned with the relationship between carotid artery atherosclerosis and its relationship with circulating inflammatory biomarkers.

Author	*N*	Patients	Markers	Results
Puz et al. [24]	95	26 asymptomatic carotid artery stenosis patients 39 symptomatic carotid artery stenosis patients 30 healthy controls	Leucocyte count, ESR, CRP, fibrinogen, TNF*α*, IL1*β*, IL-6 and IL10, and anti-cytomegalovirus IgG antibody titre	IL-6, fibrinogen, ESR, and CRP are higher in patients than in the control group. TNF*α*, IL-6, fibrinogen, number of leukocytes, monocytes, and CRP are higher in symptomatic patients than in asymptomatic patients

Debing et al. [25]	360	180 asymptomatic carotid artery stenosis patients 180 healthy controls	hs-CRP, sVCAM-1, and IL-6	hs-CRP, sVCAM-1, and IL-6 are higher in patients with stenosis than in healthy controls and correlate with the degree of stenosis

Halvorsen et al. [26]	5341	3205 asymptomatic carotid artery stenosis patients 2136 healthy controls	White blood cell count, fibrinogen, and CRP	White blood cells and fibrinogen were associated with the presence of plaque

Chapman et al. [27]	1111	Randomly selected, population-based	IL-6, hs-CRP, fibrinogen, monocyte count, and white blood cells	Only monocyte counts were associated with carotid artery plaque upon multivariate analysis

Abbas et al. [34]	181	159 high grade carotid artery stenosis patients 22 healthy controls	S100A8, S100A9, and S100A12	S100A12 levels were higher in patients with carotid atherosclerosis and highest in symptomatic patients. S100A8 and S100A9 were higher in symptomatic patients

Andersson et al. [35]	1016	Population-based survey. All individuals were 70 years of age	Apolipoprotein B/A1 ratio, OxLDL, TNF*α*, HOMA insulin resistance, leucocyte count, BCD-LDL, L-selectin	Inflammatory markers associated with plaque size

Shindo et al. [38]	58	58 asymptomatic carotid artery stenosis patients undergoing surgical or endovascular intervention	IL-6, IL1*β*, IL10, TNF*α*, E-selectin, VCAM-1, adiponectin, hs-CRP, and PTX3	PTX3 associated with histologic features of plaque vulnerability

Willems et al. [39]	391	75 asymptomatic carotid artery stenosis patients 316 symptomatic carotid artery stenosis patients	s = serum ST2	No association with features of plaque instability

Sugioka et al. [40]	102	102 asymptomatic carotid artery stenosis patients with stable coronary artery disease	Neopterin	Neopterin associated with complex plaque morphology

Pelisek et al. [41]	101	37 histologically stable asymptomatic lesions 64 histologically unstable lesions	Serum levels of MMP-1, MMP-2, MMP-3, MMP-7, MMP-8, MMP-9, TIMP-1, TIMP-2, TNF*α*, IL-1*β*, IL-6, IL-8, IL-10, and IL-12	MMP-1, MMP-7, TIMP-1, TNF*α*, and IL-8 were higher in the sera of patients bearing histological features of plaque instability

Koutouzis et al. [45]	119	57 asymptomatic patients 62 symptomatic patients	IL-6, TNF*α*, IL-1*β*, SAA, and hs-CRP	IL-6 was found higher in symptomatic patients

Garcia et al. [46]	62	36 symptomatic patients 26 asymptomatic patients	hs-CRP	hs-CRP was higher in symptomatic patients

Handberg et al. [47]	62	16 symptomatic patients <2 months 15 symptomatic patients, 2–6 months 31 asymptomatic patients	CD36	Soluble CD36 was higher in recently symptomatic patients, that is, symptoms <2 months

Edsfeldt et al. [49]	162	92 symptomatic carotid artery stenosis patients 70 asymptomatic high grade stenosis patients	suPAR	suPAR was higher in symptomatic patients and correlated with higher histological signs of inflammation

Olson et al. [50]	255	255 symptomatic carotid artery stenosis patients	suPAR(I–III), suPAR(II-III), and uPAR(I)	suPAR(I–III) and suPAR(II-III) were higher in TIAs and strokes than in amaurosis fugax

Basta et al. [51]	29	19 symptomatic carotid artery stenosis patients 10 asymptomatic carotid artery stenosis patients	sRAGE	sRAGE was higher in symptomatic patients

Abbas et al. [52]	205	182 asymptomatic carotid artery stenosis patients 23 healthy controls	MMP-7	MMP-7 was higher in patients with carotid artery stenosis than in healthy controls, with highest values in symptomatic patients

Duivenvoorden et al. [55]	130	130 patients with stable coronary artery disease	Myeloperoxidase, hs-CRP, IL-6, soluble P-selectin, soluble E-selectin, sICAM1, sVCAM-1, MMP-3, and MMP-9	Only myeloperoxidase correlated with baseline carotid artery FDG TBR in its most diseased segment

Mani et al. [56]	130	130 patients with stable coronary artery disease	IL-6, LpPLA2, apolipoprotein A-I, and hs-CRP	IL-6 correlated with baseline carotid artery FDG TBR in its most diseased segment

Rudd et al. [57]	41	41 suffering from atherosclerosis in multiple vascular districts	MMP-3, MMP-9, hs-CRP, fibrinogen, IL18, adiponectin, and PAI1	Levels of MMP-3 correlated with carotid artery FDG TBR, while a negative correlation was established between carotid TBR and PAI1

Grufman et al. [58]	160	160 patients undergoing carotid endarterectomy	hs-CRP	Blood levels of hs-CRP did not correlate with inflammatory activity within the plaque

Norata et al. [65]	156	156 healthy volunteers	LDL-RF, TGRL, and sdLDL	LDL-RF, TGRL, and sdLDL correlated with carotid IMT in healthy volunteers

Holm et al. [77]	261	28 asymptomatic carotid artery stenosis patients 31 symptomatic carotid artery stenosis patients 202 acute stroke patients, 4.4-year follow-up	FABP4	FABP4 was higher in atherosclerotic patients and associated with cardiovascular mortality in the acute stroke cohort

Mayer et al. [87]	853	853 symptomatic carotid artery stenosis patients	Neutrophils, eosinophils, basophils, monocytes, lymphocytes, and total leukocyte count	Neutrophils predicted cardiovascular mortality during 6-year follow-up

Nasr et al. [88]	60	60 symptomatic carotid artery stenosis patients	Neutrophil, lymphocytes, and monocytes counts	Neutrophils predicted the occurrence of microembolic events on transcranial Doppler ultrasound

Jurk et al. [89]	103	48 asymptomatic carotid stenosis patients 25 patients with previous stroke 30 healthy controls	Surface CD11b, P-selectin, and TSP-1 on macrophages. Platelet-monocytes aggregates	TSP-1 and P-selectin were higher on the surface of monocytes from symptomatic patients

Ritter et al. [91]	46	30 asymptomatic carotid stenosis patients 16 recently symptomatic carotid stenosis patients	P-selectin and thrombospondin expressions on platelets. Soluble P-selectin	Higher soluble P-selectin was found in patients with microembolic signals on transcranial Doppler

Sternberg et al. [92]	72	40 patients undergoing carotid endarterectomy (both symptomatic and asymptomatic) 32 healthy controls	MMP-9 and PPAR-*γ* expression levels in circulating monocytes. Several monocytes activation markers	Monocytes from patients suffering from carotid atherosclerosis displayed higher levels of activation, MMP-9, and PPAR-*γ* than in healthy controls

Martin-Ventura et al. [93]	275	70 patients undergoing carotid endarterectomy 205 healthy controls	CD74 expression in PBMC	CD74 expression was higher in PBMC of patients suffering from carotid atherosclerosis. In healthy controls it correlated with IMT

Mantani et al. [94]	700	700 subjects from the cardiovascular cohort from the Malmo study	CD19+CD40+ and CD19+CD86+ B cells count	CD19+CD86+ B cells were shown to correlate with the degree of carotid artery stenosis and the risk of stroke. CD19+CD40+ B cells were shown to predict a low risk of stroke

Baragetti et al. [97]	768	768 patients from the PLIC study	LTL	LTL was inversely correlated with IMT and the occurrence of cardiovascular events

Schulze Horn et al. [102]	3092	3092 subjects over 55 years of age from the INVADE study	hs-CRP	hs-CRP was shown to correlate with IMT

Orbe et al. [103]	400	400 healthy subjects	MMP-1, MMP-9, and MMP-10, fibrinogen, IL-6, von Willebrand factor, and hs-CRP	MMP-10 was associated with IMT

Olson et al. [104]	912	912 participants in the MESA study	CD4+CD45RA+ (naive) and CD4+CD45RO+ (memory) T cells	IMT negatively correlated with the percentage of circulating naïve T cells

Ammirati et al. [105]	183	183 free living subjects	CD3+CD4+CD45RA−CD45RO+CCR7− T effector memory cells	T effector memory cells were strongly associated with IMT

Rogacev et al. [106]	622	622 healthy volunteers	CD14++CD16−, CD14++CD16+, and CD14+CD16+ cells monocytes	CD16+ monocytes were associated with IMT

Qian et al. [111]	40	40 patients undergoing carotid endarterectomy	Soluble CD146	Soluble CD146 correlated with histologically determined plaque neovascularization

Pelisek et al. [112]	56	28 stable lesions determined by histology 28 unstable lesions determined by histology	VEGF	VEGF was higher in patients bearing plaques with histological features of instability and correlated with the degree of neovascularization

Jaipersad et al. [113]	120	40 severe (>50%) asymptomatic carotid stenosis and stable angina patients 40 patients with stable angina and stenosis of <50% 40 hypercholesterolaemic controls	CD14++ CD16−CCR2 +, CD14+CD16++ CCR2−, and CD14++ CD16+CCR2+ monocytes and their surface expression of TLR4, IL-6 receptor, and Tie2	TLR4 and Tie2 were enriched in all monocytes subsets of patients suffering from carotid stenosis. CD14++CD16−CCR2+ monocytes correlated with carotid stenosis and IMT and were associated with severe plaque neovascularization

ESR: erythrocytes sedimentation rate; CRP: C-reactive protein; TNF*α*: tumour necrosis factor *α*; IL: interleukin; sVCAM: soluble vascular cell adhesion molecule; OxLDL: oxidized lipoproteins; HOMA: homeostasis model assessment; BCD-LDL: baseline conjugated dienes of low-density lipoprotein; PTX3: pentraxin-3; MMP: matrix metalloproteinase; TIMP: tissue inhibitor of matrix proteinases; SAA: serum amyloid A; suPAR: soluble urokinase plasminogen activator receptor; sRAGE: soluble receptor of advanced glycation end products; sICAM: soluble intercellular adhesion molecule; LpPLA2: lipoprotein-associated phospholipase A2; PAI1: plasminogen activator inhibitor; LDL-RF: LDL relative floatation; TGRL: triglyceride-rich lipoproteins; sdLDL: small dense LDL; FABP4: fatty acid binding protein 4; TSP-1: thrombospondin 1; PPAR*γ*: peroxisome proliferator-activated receptor *γ*; PBMC: peripheral blood mononuclear cells; LTL: leukocyte telomere length; VEGF: vascular endothelial growth factor; TLR4: toll-like receptor 4.
